# Epidemiology of Suicide and Associated Factors in Bam: A Historical Cohort Study

**DOI:** 10.2174/0117450179398446250721063050

**Published:** 2025-07-29

**Authors:** Samane Nematolahi, Elham Isaei, Mohammad Baniasadi, Navid Reza GHasemi, Maryam Jalali, Zeinab Sarhadi, Fateme Amozegar

**Affiliations:** 1 Non Communicable Diseases Research Center, Bam University of Medical Sciences, Bam, Kerman, Iran; 2 Medical Mycology and Bacteriology Research Center, Kerman University of Medical Sciences, Kerman, Iran; 3 Mohammad Baniasadi, School of Public Health, Bam University of Medical Sciences, Bam, Iran; 4 Project Manager at Gas Company, Bam, Kerman, Iran; 5 Colorectal Research Center, Shiraz University of Medical Sciences, Shiraz, Iran; 6 Director of the Mental Health Department, Bam University of Medical Sciences, Deputy for Health, Bam, Iran; 7 Student research committee, School of nursing and midwifery, Bam university of medical sciences, Bam, Iran

**Keywords:** Suicide, Death, Epidemiology, Mental health, Suicide prevention, Antisocial behaviors

## Abstract

**Introduction:**

Suicide attempts, recognized as a significant public health concern, have been categorized among antisocial behaviors. This study aims to examine the epidemiology of suicide and its associated individual, familial, and social factors in Bam City, Iran.

**Methods:**

A historical cohort study was conducted on all recorded cases (N=3276) of suicide attempts registered in the hospital reporting system and healthcare center at Bam University of Medical Sciences from 2016 to 2022. Data were systematically extracted using a standardized checklist. Suicide attempt rates and mortality rates were calculated and presented in this study. Temporal trends in suicide mortality and attempts were analyzed using joinpoint regression.

**Results:**

The findings indicate that men reported a significantly higher suicide completion rate compared to women, with hanging being identified as the most lethal method. While overall suicide rates declined by 1.3% during the study period, a concerning 14.2% increase was observed from 2020 to 2022. Suicide rates among individuals under 35 showed a slighty increasing trend, whereas those aged 35 and older experienced a decline. Additionally, poisoning emerged as the most prevalent method across both genders.

**Discussion:**

The study underscores age-specific differences in suicide risk, highlighting the need for targeted prevention strategies. While the death rates increased among younger individuals during the pandemic, they declined for older adults, suggesting variations in vulnerability and coping mechanisms.

**Conclusion:**

Suicide prevention should be tailored to different age groups, incorporating mental health support for youth facing economic and social pressures, as well as resources for older adults. Strengthening community programs, economic assistance, and access to mental health services remain essential in reducing suicide rates across diverse demographics.

## 1 INTRODUCTION

Suicide is a complex public health issue with significant implications for individuals, families, and communities [1]. Suicide remains one of the leading causes of mortality worldwide. In 2021, it ranked among the top nine causes of death for individuals aged 10 to 64 and was the second leading cause of death among those aged 10 to 14 and 20 to 34 [2].

Nearly 800,000 individuals die by suicide each year, with 80% of these cases occurring in low- and middle-income countries [3]. “According to the World Health Organization (WHO), suicide is the intentional and conscious act of ending one's own life. A suicide attempt, by contrast, occurs when individuals deliberately harm themselves in an effort to end their lives but they survive [4].

Suicide attempts stem from a range of factors, which can be broadly classified into three primary categories: mental health disorders, social adversities, and physical health conditions [4]. Regarding social factors, the main causes include isolation, deprivation, separation, and unemployment [5]. Physical health conditions, including malignant tumors, neurological disorders, and reduced serotonin levels in the brain, have been recognized as significant contributors to suicide risk [6]. Understanding the epidemiological and demographic characteristics of individuals who engage in suicidal behavior is crucial for developing effective prevention strategies and interventions. Suicide rates differ based on race, ethnicity, age, and various contextual factors, including geographic location [7]. Individuals who have faced violence, including child abuse, bullying, or sexual violence, are at an increased risk of suicide [8]. Demographic factors, including age, gender, socioeconomic status, and geographic location, play a crucial role in shaping suicide risk. A thorough examination of these variables enables researchers and mental health professionals to gain valuable insights into the underlying determinants of suicidal behaviors [9]. A nationwide survey of 1,109,776 Korean adolescents (2005-2021) found that younger age, female gender, urban residency, smoking, alcohol use, and low socioeconomic status were significant risk factors linked to sadness and sociality [10].This study seeks to expand the existing knowledge base and inform targeted prevention initiatives across the cities served by Bam University of Medical Sciences from 2016 to 2024. By analyzing trends, demographic data, and patterns among individuals who have attempted suicide, the research aims to provide actionable insights for public health intervention.

## 2 MATERIALS AND METHODS

### 2.1 Study Design and Procedures

A historical cohort study was conducted on all recorded cases (N=3276) of suicide attempts registered in the hospital reporting system and healthcare center at Bam University of Medical Sciences from 2016 to 2022. Data were systematically extracted using a standardized checklist. Informed consent was obtained for the use of patients' data in the study. A suicide attempt or suicide is characterized as self-inflicted injury or self-poisoning, including drug overdoses exceeding therapeutic doses, leading to either a fatal or non-fatal outcome. In this study, “suicide attempt” denotes non-fatal cases, whereas “suicide” specifically refers to fatal incidents. Deaths by suicide were distinguished based on mortality records, where the cause of death was officially documented in hospital discharge summaries and forensic reports [11].

Inclusion criteria entailed all individuals of any age who presented to hospital emergency departments or healthcare centers in Bam with a documented suicide attempt or completed suicide, as registered in official medical records between January 2016 and December 2022. Cases characterized as self-inflicted injury or self-poisoning (e.g., drug overdose exceeding therapeutic doses, intentional use of lethal means) are evaluated and classified as intentional by healthcare professionals. Only cases with available hospital documentation and forensic confirmation (for fatalities) were included.

Exclusion Criteria entailed accidental poisonings or injuries, including cases involving children where intent could not be established (e.g., exploratory ingestion of medications). Cases without confirmed intent to self-harm, such as overdoses, lack supporting psychosocial or clinical evidence of suicidality. Duplicated records, incomplete documentation, or entries with missing identifiers are necessary for cohort analysis. Patients whose attempts or deaths were not classified as suicide in official records (e.g., misclassified as accidents without forensic confirmation).

Since the data in this study originates exclusively from hospital records, there is a possibility of underestimation of suicide cases, particularly in rural areas where individuals may not seek medical care or where social stigma prevents accurate reporting. Some suicide deaths may have been misclassified as accidental deaths due to limitations in hospital-based data collection. However, this study focuses on verified cases documented in hospital registries, ensuring consistency in classification based on medical assessments and official hospital records. Additionally, previous studies indicate that suicide rates in rural areas may be underestimated due to differences in reporting practices and access to mental health services [12]. We adhered to the Sex and Gender Equity in Research (SAGER) guidelines to ensure proper reporting of sex and gender-based analysis in this study.

This study was authorized by the Ethics Committee of Bam University of Medical Sciences with the number IR.MUBAM.REC.1403.011.

### 2.2 Statistical Analyses

Temporal trends in suicide death rates and suicide rates were analyzed using join point regression, allowing for up to two join points. Joinpoint Regression is a statistical method used to identify significant changes in trend patterns within a dataset. This approach allows for the detection of breakpoints where the trajectory of data shifts, enabling a more precise analysis of temporal variations. The number and location of breakpoints are determined in a data-driven manner using statistical criteria such as the Permutation Test, ensuring an optimal fit for the model. Additionally, model selection is guided by the Bayesian Information Criterion (BIC), which aids in determining the best-fitting structure while avoiding overfitting. Assumptions of the method, including independence of observations, are validated through residual analysis to ensure robustness. This technique is widely applied in epidemiological studies, economic analyses, and various fields requiring trend evaluation across segmented intervals. The changes in trend were represented as the Average Percent Change (APC) and the Average Annual Percent Change (AAPC), assuming a steady rate of change in the logarithm of the annual Incidence Rate (IR) within each segment [13]. The APC was examined by gender and age group at diagnosis from 2016 to 2022. The significance of the APC was evaluated using an asymptotic t-test with a significance level set at 5%. The Chi-square test was used to explore the association between suicide death and suicide rates and demographic variables. The analysis was performed using an SPSS version 22 and Joinpoint software (Joinpoint Regression Program (version 4.8.0.1, NCI). Given that the data was extracted from the registry, the missing values in the dataset were minimal and were excluded from the analysis.

## 3 RESULTS

Table **1** shows the association between the suicide outcome (died/alive) and demographic variables. According to this table, the relationship between suicide outcome and the gender of individuals attempting suicide was significant. The percentage of successful suicides is higher in men than in women. Additionally, the relationship between the suicide outcome and the residential area of individuals attempting suicide is not significant since the majority of individuals who have attempted suicide were observed to live in urban areas. The relationship between the suicide outcome and the method of suicide has also been examined, which is quite significant. The highest percentage of successful suicides is related to hanging, while the majority of individuals have used poisoning as a method.

Table **2** presents the frequency distribution of suicide attempts by socio-demographic factors. Poisoning is the most commonly used method among both genders. Firearms and hanging are more prevalent among men, whereas self-burning and poisoning are more frequent among women. Overall, poisoning remains the most widely used method of suicide across all individuals.

**Fig. (1):**
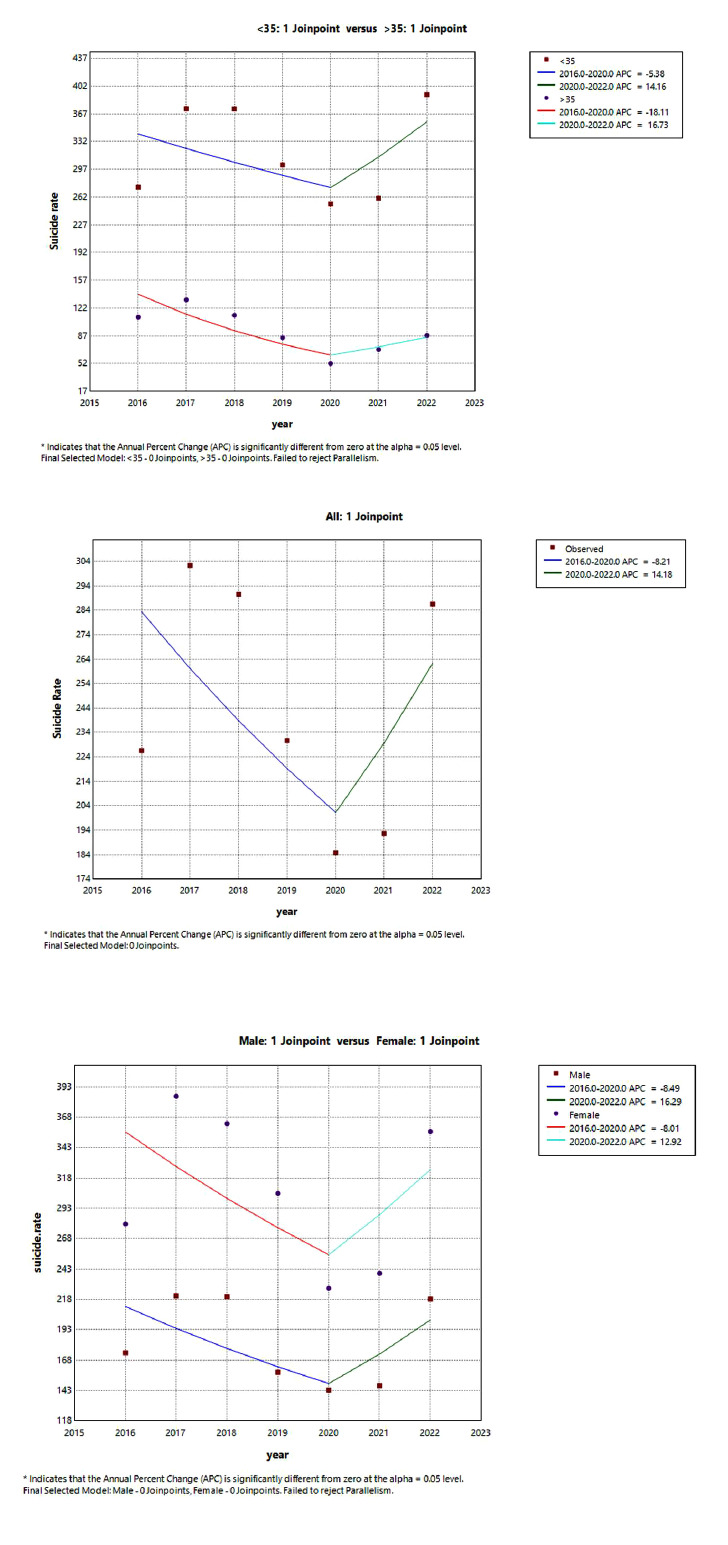
The trend in suicide rates for (**a**) suicide overall, (**b**) by gender, and (**c**) by age group in Bam between 2016 and 2022 (trend modeled with join point regression).

The analysis of suicide rates in Bam from 2016 to 2022 highlights significant trends across various age groups and genders, as summarized in the (Table **3** and Fig. **1**). This figure consists of a tree plot representing the trend in suicide rates in Bam between 2016 and 2022 with respect to (a) overall suicide, (b) gender, and (c) age group.

During 2016 to 2022, AAPC in suicide rates showed a slight decline of -1.3%. However, the APC during 2020-2022 indicated a sharp increase of 14.2%, contrasting with the preceding period (2016 to 2020), which saw a decline of -8.2%. From 2016 to 2022, the AAPC for individuals under 35 years was 0.7%. However, the APC from 2020 to 2022 showed a notable increase of 14.2%, indicating a shift in suicide trends among younger populations during this period. During the 2016–2020 period, suicide rates declined by -5.4%. Among individuals aged 35 years and older, the AAPC showed a decrease of -7.8% from 2016 to 2022. However, the APC from 2020 to 2022 exhibited a sharp increase of 16.7%, contrasting with the earlier period (2016–2020), which saw a more pronounced decline of -18.1%. Between 2016 and 2022, the AAPC for males showed a slight decline of -0.9%. However, during 2020-2022, the APC rose sharply to 16.3%, contrasting with the prior period (2016–2020), which experienced a decline of -8.5%. Similarly, the AAPC for females decreased by -1.5% over the entire period, yet the APC from 2020 to 2022 increased to 12.9%, following an earlier decline of -8.0% from 2016 to 2020.

### 3.1 Suicide Death Rates

**Fig. (2):**
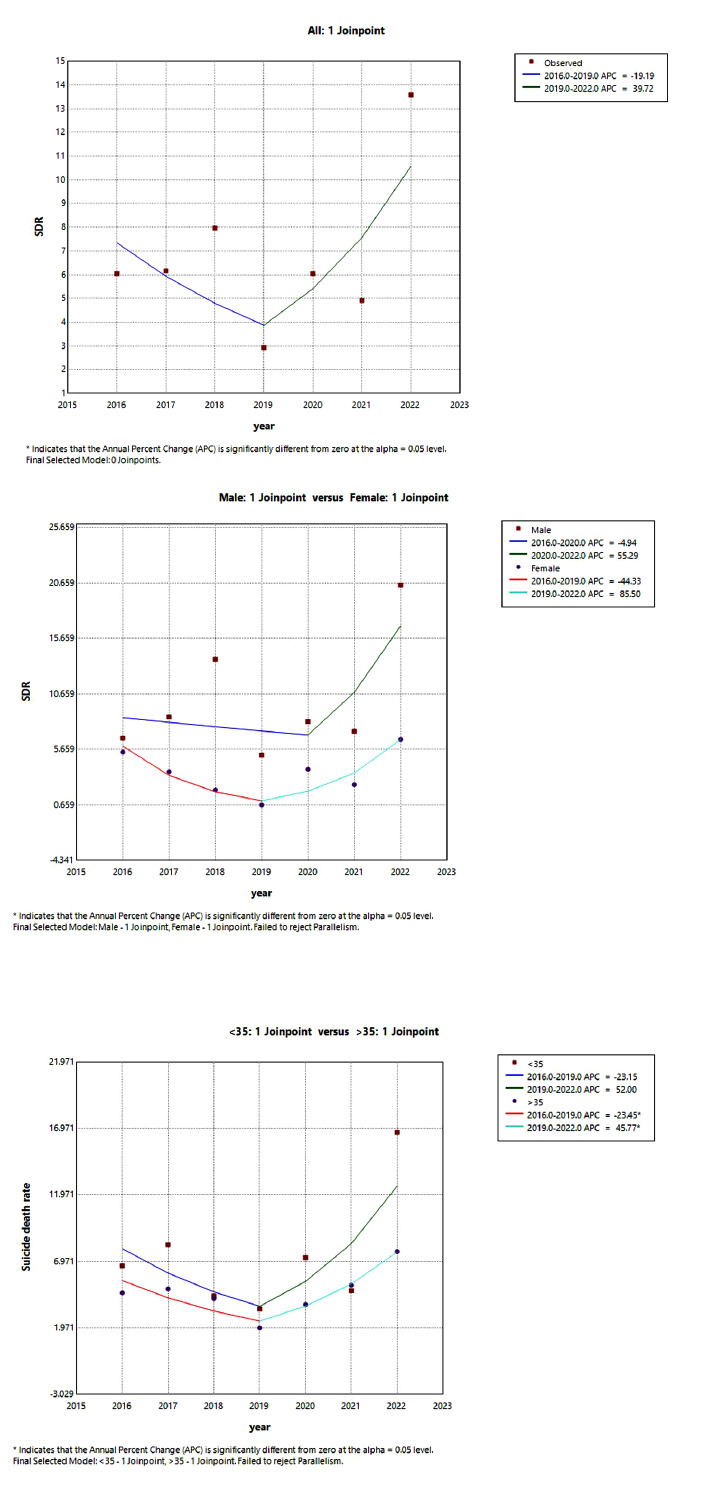
The trend in suicide death rates with respect ot (**a)** overall suicide, (**b**) gender, and (**c**) age group in Bam between 2016 and 2022 (trend modeled with join point regression).

The analysis of suicide death rates from 2016 to 2022 (Table **4** and Fig. **2**) reveals a complex trend. The overall AAPC from 2019 to 2022 showed an increase of 6.3%, the APC during this period exhibited a sharp rise of 39.7%. In contrast, the preceding period (2016 to 2019), experienced a significant decline of -19.2%, highlighting notable fluctuations in suicide mortality rates over time.

Between 2020 and 2022, the AAPC for males was 12% accompanied by a sharp APC increase of 55.3%. In contrast, the preceding period (2016-2020) showed a decline of -4.9%. For females, the AAPC from 2019 to 2022 was 1.6, with a substantial APC rise of 85.5%. However, the earlier period (2016–2019) exhibited a significant decline of -44.3% (95% CI: -84.1 to 94.8).

Among individuals under 35 years, the AAPC from 2019 to 2022 was 8.1%, with a notable APC increase of 52%. In contrast, the preceding period (2016–2019) exhibited a significant decline of -23.2%. For individuals aged 35 years and older, the AAPC was 5.6%, accompanied by an APC rise of 45.8% during 2016–2019, followed by a subsequent decline of -23.4%.

The trend in suicide death rates in Bam between 2016 and 2022 with respect to (a) overall suicide, (b) gender, and (c) age group is shown in Fig. (**2**).

## 4 DISCUSSION

Our results provide valuable insights into the complex interplay between suicide outcomes and demographic factors. Although the suicide attempt rate in Iran remains lower than the global average, it has shown an upward trend in recent years [14]. In developing countries, stressful social events are a primary driver of suicide, with family, economic, and educational struggles playing a significant role in Iran. Among women, systemic inequality and societal constraints,such as forced marriage and exposure to violence,heighten their vulnerability, often making suicide a means of escape. Furthermore, broader social factors, including poverty, unemployment, and inequality, contribute to increased suicidal tendencies, particularly among young individuals seeking relief from oppressive norms [14].

Our study underscores the strong relationship between gender and suicide outcomes, reflecting a well-documented trend: men are more likely to die by suicide than women. This aligns with previous research showing that men tend to employ more lethal methods, contributing to higher rates of completed suicides [15]. The use of highly lethal and violent suicide methods significantly increases the likelihood of a fatal outcome, with research indicating that such methods elevate the probability of a completed suicide by up to twelvefold [16]. Substance use disorders, particularly drug addiction, are the other significant contributors to the elevated suicide rates among men. The resulting mental and neurological impairments further exacerbate vulnerability, increasing the likelihood of suicidal behavior [17]. Previous studies suggest that individuals in Kerman province, which includes the city of Bam, may have higher rates of drug use compared to other regions [18]. While some studies have reported a higher rate of suicide attempts among females than males [19].

Our findings indicate that hanging is the most prevalent method among completed suicides. This reinforces its association with higher fatality rates compared to other methods. Furthermore, this trend is consistent with patterns observed in other cities in Iran as well as in countries such as Australia [20, 21].

The absence of a significant association between residential areas and suicide outcomes suggests that urban environments, despite their distinct stressors, do not inherently contribute to higher suicide rates. While numerous epidemiological studies indicate that suicide rates are generally higher in urban areas than in rural regions [13], the predominance of suicide attempts in urban settings—despite the lack of a significant correlation with outcomes—warrants further investigation into the contextual factors influencing these trends [22]. The analysis also emphasizes that poisoning is a frequently used method of suicide for both genders, which is significant due to its accessibility and the likelihood of impulsive behavior in these cases. The observed gender differences—where men are more likely to use firearms and hang, while women often choose poisoning and self-immolation—illustrate established patterns in suicidal behaviour. Previous studies report that men are more likely to use highly lethal methods than women [23]. However, there are also some exceptions to this rule [24]. Gender disparities in suicide rates in Iran are deeply influenced by sociocultural norms, including traditional gender roles, economic dependency, and legal constraints. Studies indicate that in Iran, women face higher social pressures, including forced marriage, domestic violence, and limited autonomy, which contribute to higher suicide attempt rates [25]. Conversely, men exhibit higher suicide completion rates, often linked to economic instability, substance abuse, and societal expectations of masculinity [26]. Research also highlights regional variations, where rural areas may experience underreporting due to stigma and legal concerns. Addressing these disparities requires culturally sensitive mental health interventions, improved economic opportunities, and policy reforms to mitigate gender-based suicide risks [27]. These results highlight the need to understand gender-specific risk factors and methods to create effective prevention strategies.

The temporal analysis of suicide rates in Bam from 2016 to 2022 presents a complex pattern. While the overall AAPC of -1.3% indicates a slight decline in suicide rates during this period, the sharp increase of 14.2% from 2020 to 2022 is concerning. This surge aligns with the global COVID-19 pandemic, which has been associated with worsening mental health, heightened social isolation, and economic instability,factors known to elevate suicide risk [28]. A nationwide study in South Korea analyzed sadness prevalence, counseling for sadness, and sleep patterns among 2.8 million individuals from 2009 to 2021, offering a comprehensive view of pandemic-related mental health shifts. The findings indicate that the prevalence increased during the pandemic, while counselling for sadness did not increase as expected, suggesting potential barriers to accessing mental health support. Additionally, average sleep duration, which had been declining pre-pandemic, showed an increase during the pandemic, possibly reflecting altered daily routines and psychological distress. These findings underscore the importance of monitoring long-term mental health trends and adapting intervention strategies to address evolving challenges [29]. In contrast, the significant decline of -8.2% in suicide rates from 2016 to 2020 suggests that the years preceding the pandemic may have been influenced by effective mental health interventions or evolving societal attitudes toward psychological well-being. This period may reflect increased awareness, improved access to mental health services, or policy measures that contributed to the reduction in suicide rates. Several factors may have contributed to this trend, including expanded access to mental health services, awareness campaigns, and policy improvements aimed at suicide prevention. For instance, a study by Fazel *et al.* (2019) highlighted that integrated community-based interventions led to a measurable reduction in suicide attempts by providing early psychological support and crisis intervention programs [30]. Similarly, national initiatives in suicide prevention implemented in several countries during this period, such as school-based mental health education and online support platforms, may have contributed to the declining trend seen in Iran before the pandemic [31].

The analysis of age groups shows that individuals under 35 years experienced a rise in suicide rates during the pandemic. This trend may indicate increased vulnerability among younger populations, who often face unique challenges related to social expectations, economic instability, and the stigma surrounding mental health [32]. Conversely, individuals aged 35 and older demonstrated a decline in the AAPC, indicating that this demographic may have distinct coping mechanisms or support systems that mitigate the risk of suicide.

The gender-specific trends warrant attention. While both males and females exhibited declines in the AAPC from 2016 to 2022, the substantial increases in APC during the pandemic—16.3% for males and 12.9% for females—highlight a concerning trend necessitating targeted interventions. The marked increase in suicide mortality, particularly among males, underscores the critical need for specialized mental health resources and preventive strategies tailored to this demographic. The divergent trends between suicide rates and suicide death rates in Bam reflect the multifaceted nature of mental health challenges within the population. These findings emphasize the urgency of targeted interventions aimed at mitigating the rising lethality of suicide attempts, especially among older adults and males. In Iran, societal norms and legal structures profoundly influence how suicide is reported and addressed. The topic remains heavily stigmatized, with religious doctrines and legal restrictions limiting open dialogue and precise data collection. Research suggests actual suicide rates may be underestimated, as individuals, particularly women and youth, often avoid disclosure due to potential legal penalties or community rejection [33]. Cultural norms surrounding family honor, combined with economic challenges and limited mental health resources, create substantial barriers to effective prevention measures. These findings emphasize the urgency for developing culturally adapted mental health programs, enhancing public education campaigns, and implementing policy reforms to ensure more accurate reporting and better resource allocation for at-risk populations [34]. Comprehending the underlying factors driving these trends is crucial for designing effective prevention strategies and strengthening mental health support services in the region. Further investigation is required to analyze these dynamics and inform public health initiatives. The key strengths of this study include: (a) access to national data independent of sampling constraints, and (b) nationwide data on suicide attempts, facilitating comparisons of their epidemiological characteristics with those of completed suicides.

## CONCLUSION

This study highlights the complex interplay of demographic, social, and economic factors influencing suicide risk in Iran. Gender-specific trends reveal that men face higher lethality, while women’s increased suicide attempt rates are linked to sociocultural pressures such as forced marriage and violence. Age-related patterns show that younger individuals faced heightened vulnerability during the pandemic, reinforcing the need for age-adapted interventions. Societal factors—including poverty, unemployment, and restrictive legal frameworks—play a critical role in shaping suicide trends, emphasizing the necessity for multidimensional prevention strategies.

Effective suicide prevention in Iran requires culturally sensitive approaches that enhance mental health awareness, economic support, and gender-responsive policies. Policymakers must prioritize early intervention programs, crisis resources, and expanded access to mental health services. Future research should explore the impact of policy changes, socioeconomic shifts, and regional disparities on suicide risk, ensuring data-driven strategies that effectively reduce suicide rates and improve public health outcomes.

## LIMITATION

Similar to other studies of this nature, the present study has the following limitations that may affect the interpretation and generalizability of its findings:

Hospital-Based Data – The dataset originates solely from hospital and healthcare center records, which may underestimate suicide cases that were not reported or did not result in hospitalization, particularly in rural areas with limited healthcare access. As a result of underreporting due to stigma and legal concerns, suicide remains highly stigmatized in Iran, and legal frameworks may discourage accurate reporting. This could lead to underreporting, especially among women and youth, who may fear legal consequences or community rejection.

Limited Access to Psychological and Social Variables – This study lacks data on mental health diagnoses, social determinants such as substance abuse, drug addiction, and alcohol consumption, as well as economic indicators that may impact suicide risk. These missing variables limit a comprehensive analysis of contributing factors.

Regional Constraints – Since the study is based on data from Bam, findings may not be fully generalizable to other areas with different economic conditions, social structures, or access to healthcare services.

Potential Misclassification – Some suicide deaths may have been misclassified as accidental deaths due to limitations in hospital-based coding systems, affecting the accuracy of suicide trends, particularly in rural areas.

Data Exclusion Due to Missing Values – Although the registry-based dataset had minimal missing values, any cases with missing data were excluded from the analysis, which may slightly impact the final estimates.

## Figures and Tables

**Table 1:** Association between suicide outcomes (suicide attempts and completed suicides) and demographic variables.

Variable	Group	Died	Live	*P* value*
Gender	Male	85 (5%)	1605 (95%)	< 0.0001
	Female	28 (1%)	2758 (99%)	
Marital status	Single	51(2.7%)	1832 (97.3%)	0.0001
	Married	50 (2%)	2468 (98%)	
	Divorced	3 (33.3%)	6 (66.7%)	
	Widowed	0 (0%)	3 (100%)	
	Missing	9 (14.3%)	54 (85.7%)	
Job	Student	24 (2.5%)	938 (97.5%)	< 0.0001
	Soldier	4 (16.7%)	20 (83.3%)	
	Housewife	10 (0.7%)	1339 (99.3%)	
	Worker	11 (4.1%)	259 (95.9%)	
	Jobless	23 (5.1%)	432 (94.9%)	
	Other	19 (2.9%)	647 (97.1%)	
Residence area	Rural	58 (3%)	1870 (97%)	0.073
	Urban	55 (2.16%)	2493 (97.84%)	
Suicide method	Cold weapon	2 (4.5%)	42 (95.5%)	0.0001
	Firearms	9 (100%)	0 (0%)	
	Self-burning	9 (60%)	6 (40%)	
	Hanging	55 (70.5%)	23 (29.5%)	
	Poisoning	33 (8.0%)	4246 (99.2%)	
	Other	5 (9.8%)	46 (90.2%)	

*Chi-square Test

**Table 2:** Frequency distribution of suicide attempts by socio-demographic characteristics.

Variable	Group	Male	Female	*P* value
Marital status	Single	989 (58.5%)	894 (32.1%)	< 0.0001
	Married	669 (39.6%)	1849 (66.4%)	
	Divorced	3 (0.2%)	34 (1.2%)	
	Widowed	0	3 (0.1%)	
	Missing	29 (1.7%)	6 (0.2%)	
Job	Student	380 (27.3%)	582 (24.9%)	< 0.0001
	Soldier	24 (1.7%)	0	
	Housewife	0	1349 (57.8%)	
	Worker	248 (17.8%)	22 (0.9%)	
	Jobless	415 (29.8%)	40 (1.7%)	
	Other	324 (23.3%)	342 (14.6%)	
Residence area	Rural	713 (37%)	1215 (63%)	0.352
	Urban	977 (38.3%)	1571 (61.7%)	
Suicide method	cold weapon	20 (45.5%)	24 (54.4%)	< 0.0001
	Firearms	8 (88.9%)	1 (11.1%)	
	Self-burning	3 (20%)	12 (80%)	
	Hanging	67 (85.9%)	11 (14.1%)	
	Poisoning	1564 (36.6%)	2715 (63.4%)	
	Other	28 (54.9%)	23 (45.1%)	

**Table 3:** Trends in suicide rates in Bam (2016–2022), analyzed by age group and gender.

2016-2022	Trend 2		Trend 1		-
AAPC (95% CI)	APC (95% CI)	Period	APC (95% CI)	Period	
-1.3 (-19.3-20.8)	14.2 (-62.9,251.5)	2020-2022	-8.2 (-35.7,31)	2016-2020	Overall
Age group					
0.7 (-17.6,23.1)	14.2(-62.6,248.7)	2020-2022	-5.4(-33.5,34.7)	2016-2020	< 35 years
-7.8 (-23.4,10.8)	16.7(-58.2,225.8)	2020-2022	-18.1 (-40.8,13.3)	2016-2020	>= 35 years
Gender					
-0.9 (-20.5,23.7)	16.3 (-66, 298.2)	2020-2022	-8.5 (-38,35.1)	2016-2020	Male
-1.5 (-19,19.7)	12.9 (-61.9,234.9)	2020-2022	-8 (-34.8,29.7)	2016-2020	Female

**Abbreviations:** APC annual percentage change, AAPC average annual percent change, CI confidence interval.

**Table 4:** Trends in suicide death rates in Bam (2016–2022), analyzed by age group and gender.

2016-2022	Trend 2		Trend 1		-
AAPC (95% CI)	APC (95% CI)	Period	APC (95% CI)	Period	
6.3 (-25.3,51.3)	39.7 (-53.3,318.1)	2019-2022	-19.2 (-73,141.8)	2016-2019	Overall
Age group					
8.1 (-36.3,83.5)	52 (-70.6,686.3)	2019-2022	-23.2(-8.1,297.5)	2016-2019	< 35 years
5.6 (-1.1,12.8)	45.8*(18.8,78.9)	2016-2019	-23.4* (-37.6, -6)	2016-2019	>= 35 years
Gender					
12(-36,95.9)	55.3 (-93.1, 3391.3)	2020-2022	-4.9 (-64.5,154.4)	2016-2020	Male
1.6 (-32.1,52.1)	85.5 (-47,549)	2019-2022	-44.3 (-84.1,94.8)	2016-2019	Female

**Abbreviations:** APC annual percentage change, AAPC average annual percent change, CI confidence interval. *The annual percent changes (APC) were significantly different from 0 for a specific trend (P-value < 0.05).

## Data Availability

The data that support the findings of this study are available from the corresponding author, [M.J.], on special request.

## LIST OF ABBREVIATIONS

WHO: World Health Organization
BIC: Bayesian Information Criterion
APC: Average Percent Change
AAPC: Average Annual Percent Change
IR: Incidence Rate

## STANDARDS OF REPORTING

STROBE guidelines were followed.
